# Non-overlapping and Inverse Associations Between the Sexes in Structural Brain-Trait Associations

**DOI:** 10.3389/fpsyg.2019.00904

**Published:** 2019-04-24

**Authors:** Daphne Stam, Yun-An Huang, Jan Van den Stock

**Affiliations:** ^1^Laboratory for Translational Neuropsychiatry, Department of Neurosciences, KU Leuven, Leuven, Belgium; ^2^Geriatric Psychiatry, University Psychiatric Center KU Leuven, Leuven, Belgium; ^3^Brain and Emotion Laboratory, Maastricht University, Maastricht, Netherlands

**Keywords:** sex, temperaments, voxel-based morphometry, brain-trait association, full factor model

## Abstract

Personality reflects the set of psychological traits and mechanisms characteristic for an individual. The brain-trait association between personality and gray matter volume (GMv) has been well studied. However, a recent study has shown that brain structure-personality relationships are highly dependent on sex. In addition, the present study investigates the role of sex on the association between temperaments and regional GMv. Sixty-six participants (33 male) completed the Temperament and Character Inventory (TCI) and underwent structural magnetic resonance brain imaging. Mann-Whitney *U* tests showed a significant higher score on Novelty Seeking (NS) and Reward Dependence (RD) for females, but no significant group effects were found for Harm Avoidance (HA) and Persistence (P) score. Full factor model analyses were performed to investigate sex-temperament interaction effects on GMv. This revealed increased GMv for females in the superior temporal gyrus when linked to NS, middle temporal gyrus for HA, and the insula for RD. Males displayed increased GMv compared to females relating to P in the posterior cingulate gyrus, the medial superior frontal gyrus, and the middle cingulate gyrus, compared to females. Multiple regression analysis showed clear differences between the brain regions that correlate with female subjects and the brain correlates that correlate with male subjects. No overlap was observed between sex-specific brain-trait associations. These results increase the knowledge of the role of sex on the structural neurobiology of personality and indicate that sex differences reflect structural differences observed in the normal brain. Furthermore, sex hormones seem an important underlying factor for the found sex differences in brain-trait associations. The present study indicates an important role for sex in these brain structure-personality relationships, and implies that sex should not just be added as a covariate of no interest.

## Introduction

Some people are almost constantly looking for new challenges, while others choose to stick to old habits. There is a large diversity in the way people behave and how they think. This diversity can be explained by personality, a set of psychological traits and mechanisms characteristic for an individual (Larsen and Buss, 2010). It is well known that personality traits are subject to sex differences. For instance, females typically show higher agreeableness and neuroticism compared to males (Chapman et al., 2007; Weisberg et al., 2011). Little is known about the neurobiology that is associated with sex differences in personality traits. However, there are striking differences between the sexes in the neural basis of emotional processes (Kret and De Gelder, 2012), in the relationship between narcissistic personality and regional grey matter volume (GMv; Yang et al., 2015) and findings implicate structural differences as a partial explanation for sex differences in antisocial personality (Raine et al., 2011). A recent study of Nostro et al. (2016) showed that brain structure-personality associations are highly dependent on sex. They used the NEO Five Factor Inventory (NEO FFI) to measure personality and found no significant associations between the NEO FFI (Costa and McCrae, 1992b) and regional (GMv) for the combined (males and females) sample. However, they did find sex-specific associations. Interestingly, significant associations with GMv were detected only in males. For neuroticism negative correlations were found for GMv of parieto-occipital sulcus/cuneus, left fusiform gyrus/cerebellum, and right fusiform gyrus. Positive correlations were found between conscientiousness and GMv of left precuneus and parieto-occipital sulcus. Also a positive correlation was found between extraversion and GMv precuneus/parieto-occipital sulcus, thalamus, left fusiform gyrus/cerebellum, and right cerebellum.

The present study addresses sex differences in the neurobiology of temperaments which are based on the psychobiological personality account of Cloninger et al. (1993). Temperaments are regarded to be heritable and homogeneous, stable over time, emerge early in life, and independent of each other (Cloninger, 1986; Cloninger et al., 1993; Heath et al., 1994; Stallings et al., 1996; Comings et al., 2000; Larsen and Buss, 2010). The Temperament and Character Inventory (TCI) assess these temperaments (Cloninger et al., 1993). The TCI contains four temperament scales: (1) novelty seeking (NS); (2) harm avoidance (HA); (3) reward dependence (RD); and (4) persistence (P) (Cloninger et al., 1993). These temperament scales can be further subdivided into different subscales: NS can be divided into exploratory excitability (NS1), impulsiveness (NS2), extravagance (NS3), and disorderliness (NS4); HA is composed of anticipatory worry (HA1), fear of uncertainty (HA2), shyness (HA3), and fatigability (HA4); RD can be divided into sentimentality (RD1), social attachment (RD2), and dependency (RD3); the temperament P is not further divided (Cloninger et al., 1993).

Several studies have investigated the associations between temperaments and regional GMv (Iidaka et al., 2006; Gardini et al., 2009; Picerni et al., 2013; Laricchiuta et al., 2014; Stam et al., 2018). However, sex is a variable that is typically statistically controlled for and little is known about its effect on temperament-brain associations.

In the current study, we try to answer the question “how does sex affect the association between temperaments and regional GMv?” In order to answer this question, we investigate the interaction between sex and temperaments (NS, HA, RD, and P) on regional GMv. Not much is known about the relation between sex and the association between temperaments and regional GMv. The study of Nostro et al. (2016) showed a positive correlation between extraversion and GMv precuneus/parieto-occipital sulcus, thalamus, left fusiform gyrus/cerebellum, and right cerebellum, in males. As extraversion is a trait known to be linked with NS (Gocłowska et al., 2018), we expect to find comparable results. The aim of the current study is to reveal distinct and common effects between the sexes in associations between regional GMv and temperaments. Furthermore, sex-specific associations between personality and risk for neuropsychiatric disorders have been reported, including eating disorders (Gual et al., 2002; Krug et al., 2009) and mood disorders (Costa and McCrae, 1992a; Afifi, 2007). A better understanding of the neurobiology of sex differences in personality temperaments may hold benefits for development of sex-specific treatments of those disorders.

## Materials and Methods

This study was approved by the Ethical Committee of University Hospitals Leuven. All subjects gave written informed consent in accordance with the Declaration of Helsinki.

### Participants

Sixty-six healthy subjects participated, 33 males (mean age ± SD = 38 ± 13 years, range 21–75) and 33 females (mean age ± SD = 36 ± 11 years, range 21–65)^1^. Mann-Whitney *U* tests showed that no significant sex differences were detected for age (*P* = 0.568). The sample was a non-clinical population composed of three subgroups to increase the variability of the loadings on personality scales: (1) Fourteen participants with premanifest Huntington’s disease [21% (50% male)], (2) Eighteen gene-negative controls from Huntington’s disease families [27% (50% male)], (3) thirty-four healthy controls [52% (50% male)]. We included premanifest Huntington’s disease subjects, referring to the absence of motor symptoms in combination with a positive mutation status. In addition, a radiologist evaluated the structural scans and there were no abnormalities at the individual level. As the main question of the current article focusses on sex differences, observing possible group differences of the three subgroups falls outside the scope of this article. The different subgroups are merely added for methodological reason (increasing the variability in the dataset).

### Temperament and Character Inventory

Temperament and character inventory is a questionnaire for measuring seven domains of personality and consists of 240-dichotomous items. The seven domains are divided in three character scales (self-directedness, cooperativeness, and self-transcendence) and four temperament scales (NS, HA, RD, and P). A validated Dutch translation was used with reasonable to good psychometric internal consistency (Cronbach’s α range, 0.64–0.87). It was validated in a representative sample of Dutch individuals (*n* = 1034) (version 1.3; Datec Psychological Tests, Leiderdorp, Netherlands). NS reflects enthusiasm, impulsivity, and reward-sensitivity (e.g., “I like to explore new ways to do things”); HA is related to acting with caution and passive avoidance behavior (e.g., “I often feel tense and worried in unfamiliar situations, even when others feel there is little to worry about”); RD is associated with responsiveness to signals of reward (e.g., “I like to please other people as much as I can”) and P indicates motivation without direct external reward (e.g., “I am more of a perfectionist than most people”) (Cloninger, 1986; Cloninger et al., 1993; Larsen and Buss, 2010; Laricchiuta et al., 2014).

### MRI Acquisition

Neuroimaging was performed on a 3T MRI scanner. A high-resolution T1-weighted anatomical image (voxel size: 0.98 mm × 0.98 mm × 1.20 mm) was acquired on a single 3T Philips Achieva system equipped with a 32 channel head coil using a 3D turbo field echo sequence (TR:9.6 ms; TE:4.6 ms; matrix size:256 × 256; 182 slices).

### Structural Data Analysis

Data was analyzed using CAT12, a Computational Anatomy Toolbox^2^ (Gaser and Kurth, 2017) running under SPM12^3^ and MATLAB (R2016b). In order to investigate the role of sex in the association between regional GMv and temperaments, we performed voxel-based morphometry (VBM). Preprocessing consisted of normalization to MNI space, tissue classification (segmentation) into GM, white matter (WM), and cerebrospinal fluid (CSF), and bias correction of intensity non-uniformities. The amount of volume changes due to spatial registration were scaled, in order to retain the original local volumes (modulating the segmentations). The modulated images were smoothed using a 12 mm × 12 mm × 12 mm full-width at half-maximum Gaussian kernel.

### Statistical Analysis

In order to investigate sex differences in both the main scales (NS, HA, RD, and P) as in the subscales (NS1, NS2, NS3, NS4, HA1, HA2, HA3, HA4, RD1, RD2, RD3, and P) statistical tests were preceded by a normality check on the distributions of the respective residuals by means of Shapiro-Wilk test. In case normality could not be assumed, non-parametric tests were performed. For the purpose of uniformity of analyses, we performed parametric tests or non-parametric tests on all behavioral data.

To investigate sex differences in brain-temperament associations, we first performed a full factorial model analysis on the voxelwise GMv of the total sample, to observe the interaction effect between sex and the temperament scores (NS, HA, RD, and P). Sex was included as factor and the temperament scores as interaction with the factor. Age and total intracranial volume (TIV) were included as variables of no interest.

Eight contrasts were performed: for every temperament scale, we investigated the male and female association. In every analysis we added the remaining temperament scores as variables of no interest, in order to maximize the specificity of the results of a single temperament (as it controls for the association that is contained by the other temperaments).

Secondly, multiple regression analyses were performed on the smoothed GM-images for males and females separately. The four temperament scores (NS, HA, RD, and P) were entered as regressors in a single model, in addition to age, and TIV, which were included as variables of no interest. In total eight contrasts were performed on the male data and eight contrasts on the female data.

To investigate any overlap between the sex-selective results, we inclusively masked the results of the regression analysis of both groups. The statistical threshold was set at a *P*_height_ < 0.001 (*k* = 10) in combination with *P*_height_ < 0.05 FWE-corrected following Small Volume Correction.

Anatomic labeling of significant clusters was performed using xjView^4^ and clusters were visualized using MRICron^5^.

## Results

**FIGURE 1:**
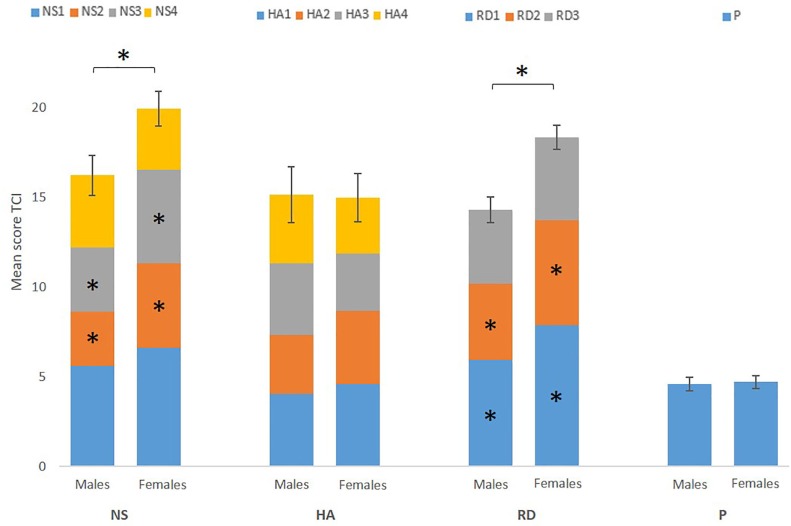
Mean score of the four temperament scores [novelty seeking (NS), harm avoidance (HA), reward dependence (RD), and persistence (P)] and each subscale (NS1, NS2, NS3, NS4, HA1, HA2, HA3, HA4, RD1, RD2, RD3, and P) for males and females separately. Error bars represent standard error of the total score (NS, HA, RD, and P). The results show a significant higher score on NS and RD for females, driven by NS2, NS3, RD1, and RD2. ^∗^Marks significance at *p* < 0.05.

Shapiro-Wilk test showed that residuals of NS, HA, RD, and P were normally distributed (*P* > 0.108). The residuals of the different subscales, however, were not normally distributed. For the purpose of uniformity of analyses, we performed non-parametric tests on all behavioral data. Mann-Whitney *U* tests showed a significantly higher score on NS (*p* = 0.02) and RD (*p* < 0.001) for females, but no significant group effects were found for the HA and P score (*P* > 0.445). Furthermore, Mann-Whitney *U* tests showed sex differences between the subscales (Figure 1).

### Interaction Between Sex and Temperaments in Voxel-Wise GMv

**FIGURE 2:**
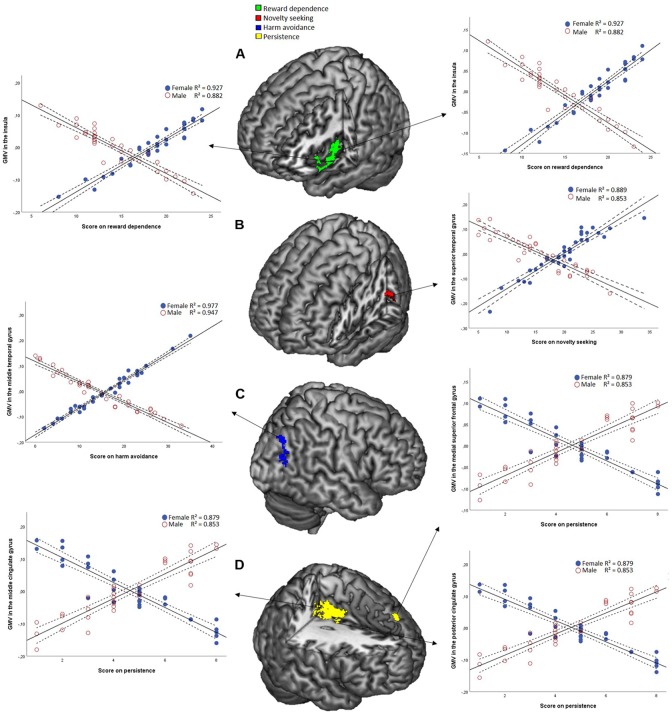
Full factorial model results obtained at a statistical threshold of *P*_height_ < 0.001 (*k* = 10), combined with SVC, FWE-corrected at cluster level. The results are shown at a significance level of *P* = 0.05 and are overlaid on a canonical 3-dimensional–rendered MRI brain template with a cut-out. **(A)** In the middle a statistical map displaying the sex-temperament interaction effects on GMv for reward dependence. Left and right the partial correlation (Female: *r* = 0.963, Male: *r* = –0.939) between GMv in the insula as a function of reward dependence. **(B)** In the middle a statistical map displaying the sex- temperament interaction effects on GMv for novelty seeking. Right a scatterplot showing the partial correlation (Female: *r* = 0.943, Male: *r* = –0.925) between GMv in the superior temporal gyrus as a function of novelty seeking. **(C)** In the middle a statistical map displaying the sex-temperament interaction effects on GMv for harm avoidance. Left a scatterplot showing the partial correlation (Female: *r* = 0.989, Male: *r* = –0.973) between GMv in the middle temporal gyrus as a function of harm avoidance. **(D)** In the middle a statistical map displaying the sex-temperament interaction effects on GMv for persistence. Left a scatterplot showing the partial correlation (Female: *r* = –0.938, Male: *r* = 0.924) between GMv in the middle cingulate gyrus as a function of persistence. Right a scatterplot showing the partial correlation (Female: *r* = –0.938, Male: *r* = 0.924) between GMv in the medial superior frontal gyrus as a function of persistence and the partial correlation (Female: *r* = –0.938, Male: *r* = 0.924) between GMv in the posterior cingulate gyrus as a function of persistence.

To investigate the role of sex in the association between regional GMv and temperaments, we ran a full factorial model on the smoothed GM images. This revealed interactions between sex and temperaments. The results are presented in Table 1. Males and females show opposite associations between NS and GMv in the superior temporal gyrus (females show positive association; *t* = 4.96, *p* = 0.001), for HA in the middle temporal gyrus (females show positive association; *t* = 4.56, *p* = 0.003), for RD in the insula (females show positive association; *t* = 4.42, *p* = 0.003 and *t* = 3.66, *p* = 0.001), for P in the posterior cingulate gyrus (females show a negative association; *t* = 4.72, *p* = 0.006), the medial superior frontal gyrus (females show a negative association; *t* = 4.62, *p* = 0.006), and the middle cingulate gyrus (females show a negative association; *t* = 4.56, *p* = 0.001). See Figure 2.

**Table 1:** Sex-temperament interaction effects in voxel-wise GMv.

	Area	*P*	R/L	#Voxels	*Z*-value	*t*-value	Coordinates		
							
							x	y	z
NS	Superior temporal gyrus	0.001	R	12	4.51	4.96	56	-44	18
HA	Middle temporal gyrus	0.003	L	10	4.56	5.04	-50	-74	23
RD	Insula	0.003	R	11	4.42	4.09	35	-8	9
	Insula	0.001	R	15	3.66	3.45	35	17	-14
P	Posterior cingulate gyrus	0.006	R	12	4.72	5.25	5	-36	29
	Medial superior frontal gyrus	0.006	L	12	4.62	5.11	11	44	48
	Middle cingulate gyrus	0.001	L	18	4.56	5.03	0	-32	36


*Overview results full factorial model [*P*_*height*_ < 0.001 (*k* = 10), combined with SVC, FWE-corrected at cluster level], observing the role of sex for GMv associations with temperament traits of the TCI; novelty seeking (NS), harm avoidance (HA), reward dependence (RD), and persistence (P). Coordinates refer to MNI-space.*

### Within-Sex Correlation Between Temperaments and Voxel-Wise GMv

To investigate the correlation between temperaments and voxel-wise GMv for males and females separately, we performed two separate multiple regression analyses on the smoothed GM-images. The results are shown in Table 2. To investigate any overlap between the sex-selective results, we inclusively masked the results of the regression analysis of both groups. No overlap was observed.

**Table 2:** Within-sex correlation between temperaments and voxel-wise GMv.

	Area	*r*	*P*	R/L	#Voxels	*Z*-value	*t*-value	Coordinates		
								
		+/-						x	y	z
NS Female	Caudate nucleus	+	<0.001	R	22	4.24	5.16	3	9	-6
	Thalamus	+	0.002	L	10	3.68	4.27	-45	-62	50
	Angular gyrus	+	0.001	L	13	3.55	4.07	-18	-26	11
	Anterior cingulate sulcus	-	<0.001	L	20	3.60	4.14	0	32	11
	Mid frontal gyrus	-	<0.001	L	30	4.32	5.29	-35	2	62
NS Male	Rolandic operculum	+	<0.001	R	69	4.28	5.13	53	5	2
	Precentral gyrus	+	<0.001	R	21	4.22	5.22	30	-9	56
HA Female	Inferior frontal gyrus	+	0.002	L	14	4.20	5.09	-18	32	-24
	Fusiform gyrus	+	0.001	L	16	4.31	5.28	-44	-65	-18
	Superior temporal sulcus	+	0.001	R	15	3.91	4.62	60	2	-5
	Superior temporal sulcus	+	0.002	L	13	4.39	5.40	-65	-39	11
	Mid temporal sulcus	+	0.004	L	11	4.01	4.78	-59	-51	14
	Supramarginal gyrus	+	<0.001	R	19	4.04	4.82	63	-24	33
	Cerebellum (L9)	-	<0.001	R	15	3.80	4.45	8	-57	-59
HA Male	Rolandic operculum	+	<0.001	R	25	4.14	4.99	53	5	2
	Mid cingulate sulcus	+	<0.001	R	17	3.51	4.02	5	5	30
RD Female	Cerebellum Crus1	+	<0.001	L	30	3.87	4.56	-33	-78	-29
	Cerebellum Crus1	+	0.001	L	15	3.78	4.42	-24	-84	-27
	Cerebellum Crus1	+	0.003	L	12	3.54	4.06	-21	-80	-27
	Frontal inferior orbital gyrus	+	0.001	L	17	3.96	4.65	-24	24	-20
	Insula	+	0.004	R	11	4.15	5.01	35	-9	11
	Fusiform gyrus	-	0.001	L	15	3.49	3.99	-39	-60	-21
	Insula	-	0.002	L	11	3.58	4.11	-38	-11	-6
	Insula	-	0.002	L	11	3.78	4.41	-36	-30	21
RD Male	-									
P Female	Cerebellum (L7b)	-	<0.001	L	161	4.77	6.12	-44	-53	-53
	Parahippocampal gyrus	-	<0.001	L	35	4.28	5.22	-18	-26	-20
	Inferior frontal gyrus	-	<0.001	R	53	5.04	6.66	21	12	-18
	Thalamus	-	<0.001	R	33	4.19	5.07	11	-30	9
	Posterior cingulate gyrus	-	<0.001	R	56	4.46	5.54	5	-51	11
	Parahippocampal gyrus	-	0.001	R	17	4.45	5.53	17	-41	-9
	Inferior Frontal gyrus	-	<0.001	L	24	4.13	4.98	-29	38	-14
P Male	-									


*Overview Multiple regression results [*P*_*height*_ < 0.001 (*k* = 10), combined with SVC, FWE-corrected at cluster level], investigating GMv associations with temperaments of the TCI; novelty seeking (NS), harm avoidance (HA), reward dependence (RD), and persistence (P) for males and females separately. Coordinates refer to MNI-space. L9, lobe 9; L7b, lobe 7b.*

## Discussion

In the current study, we investigated sex differences in temperament-brain associations, as well as shared temperament-brain associations between the sexes.

### Sex Differences in Temperament Traits

We observed significant sex differences in NS and RD between males and females. For both temperaments, females had a significantly higher score than males.

Extraversion, is a trait of the Big Five that is known to be linked with NS (Gocłowska et al., 2018). A study by Weisberg et al. (2011) observed significantly higher overall extraversion score in females. Furthermore, individuals scoring high with respect to NS tend to be enthusiastic, impulsive, and NS is known to be linked to the neurotransmitter dopamine (Cloninger, 1987; Larsen and Buss, 2010). Previous research has shown that females score higher in enthusiasm (Costa et al., 2001; Weisberg et al., 2011) and that estradiol, the female sex hormone, modulates mesolimbic dopamine systems and so affects motivated behaviors (Yoest et al., 2014). On the other hand, no associations between the total scores of NS and total testosterone, the male sex hormone, have been found (Tsuchimine et al., 2015). Previous studies have looked at sex differences in a previous version of the TCI, the Tridimensional Personality Questionnaire (TPQ) (Cloninger et al., 1991). However, these studies found conflicting findings on NS score and impulsivity when looking at sex differences (Reynolds et al., 2006; Mitchell and Potenza, 2015). A possible explanation for these conflicting findings on impulsivity and NS, may be that females show fluctuating levels of impulsivity due to the menstrual cycle and changing estrogen levels (Weafer and de Wit, 2014). Furthermore, when looking at the different subscales of NS, we found that the impulsiveness (NS2) (*p* = 0.029) and the extravagance (NS3) (*p* = 0.005) dimensions of NS specifically drive the significant sex differences in NS. A study using the TPQ (Cloninger, 1987) also found a significantly higher score for females on NS3 (Zohar et al., 2001). They also found a positive correlation between NS3 and RD, the second temperament where we found a significant higher score for females than for males.

Reward dependence has been shown to be linked with norepinephrine, previous research has already shown that through the central nervous system estrogen can modulate noradrenergic neurotransmission (Vega-Rivera et al., 2013). Furthermore, our findings on RD are in line with previous research (Cloninger et al., 1991; Zohar et al., 2001) and showed that for RD the significant sex difference was driven by sentimentality (RD1) (*p* < 0.001) and attachment (RD2) (*p* = 0.010). RD is often described as inter-personal sensitivity and sociability. Generally, females focus more on interpersonal relationships, score higher on attachment, warmth and empathy (Zohar et al., 2001; Weisberg et al., 2011; Weafer and de Wit, 2014) and are more concerned with the opinion of others in social tasks than males (Cloninger et al., 1991; Vega-Rivera et al., 2013). Males tend to focus more on individuality and achievement (Sato and McCann, 1998). These results provide support for the higher score for females on RD.

In summary, we found significantly higher scores for females on NS and RD. These results indicate that mainly for the temperaments linked to sociability and attachment, we find a significant higher score for females.

### Interaction Between Sex and Temperaments on Voxel-Wise GMv

We found opposite associations in both groups between GMv in the superior temporal gyrus and NS, with a positive association for females. Previous research found a significant positive correlation between NS and glucose metabolism in the superior temporal gyrus (Hakamata et al., 2006), which is a region that is linked with impulsivity and social cognition (Horn et al., 2003; Grecucci et al., 2013). Furthermore, a previous study showed that females mainly have more GM percentage in the superior temporal gyrus than males (Schlaepfer, 1995). A study looking at the difference between pre- and post-menopausal females, showed a decrease in GMv in post-menopausal females. The GMv was also found to be positively correlated to estradiol, the major female sex hormone (Kim et al., 2018). In contrast with our hypothesis we did not find any results for NS that were comparable to the study of Nostro et al. (2016). A possible explanation for this discrepancy may relate to the methods (Multiple regression analysis vs. full factorial model analysis). Alternatively, the similarity between NS and extraversion may be limited.

Secondly, we observed inverse associations in both groups between GMv in the insula and RD, with a positive association in females. RD is associated with responsiveness to signals of reward, the insula is known to play a part in the additional reward-sensitive brain areas (O’Doherty et al., 2002; Kirsch et al., 2003). Research shows that females show more GMv in the right insula (Ruigrok et al., 2014) and estrogen is found to excite neurons in the insula (Saleh et al., 2004). The insula is part of the limbic system. Previous research has shown that females have a larger limbic volume (Goldstein et al., 2001; Zaidi, 2010). It has been proposed that due to a larger limbic brain females are better in touch with their emotions and can better connect to others (Zaidi, 2010).

Thirdly, we found opposite associations between HA and GMv in the middle temporal gyrus. There are contradictory findings about the link between HA and the middle temporal gyrus (Hakamata et al., 2006; Iidaka et al., 2006). As previous studies have shown both positive (Iidaka et al., 2006) and negative correlations (Hakamata et al., 2006) between HA and the middle temporal gyrus. The middle temporal gyri is also known to be linked to social cognition (Grecucci et al., 2013). Furthermore, we did not find a significant sex difference in HA score.

P is the only temperament with opposite associations for which we found a positive association for males. We did not find a sex difference for score in P. P indicates motivation without direct external reward (Cloninger et al., 1993). The medial superior frontal gyrus and the cingulate gyri are areas known to be involved in cognitive control and motivation (Heilbronner et al., 2011; Bahlmann et al., 2015).

In summary, the results show that GMv of the superior temporal gyrus, middle temporal gyrus, and insula show a positive association between temperaments and regional GMv in females and a negative association in males, while a positive association in males and a negative association in females was observed between P and regional GMv in the posterior cingulate gyrus, medial superior frontal gyrus, and middle cingulate gyrus.

### Within-Sex Correlation Between Temperaments and Voxel-Wise GMv

We found non-overlapping sex-specific topographic patterns in temperament-brain associations. These results suggests that the structural neurobiology underlying personality is to a high degree sex-specific and our results are in line with the study of Nostro et al. (2016), who only found sex-specific associations. Our results support their hypothesis that brain structure-personality associations are highly dependent on sex and this may be attributable to hormonal interplays.

### Clinical Implications

Different temperaments from the TCI have been linked to several neuropsychiatric disorders; NS is known to be correlated with drug addiction (Bardo et al., 1996; Lin et al., 2015; Vanhille et al., 2015), tobacco abuse (Palmer et al., 2013), and depression (Duclot and Kabbaj, 2013). NS and HA are both linked to pathological gambling (Kim and Grant, 2001; Nordin and Nylander, 2007) and alcohol abuse (Palmer et al., 2013; Wennberg et al., 2014). However, the risk for these neuropsychiatric symptoms differs between males and females. For example, studies show that males have a higher risk for developing an alcohol or gambling addiction (Engwall et al., 2004; Nolen-Hoeksema and Hilt, 2006). Our results support the importance of sex in the neurobiology of these disorders.

### Limitations

A limitation of the current study is the small sample size. However, the different subgroups that constituted the sample presumably increased the variability of the dataset and benefited the statistical power. Furthermore, we used a statistical threshold [*P*_height_ < 0.001 (*k* = 10) in combination with *P*_height_ < 0.05 FWE-corrected following Small Volume Correction]. However, as the current study is an exploratory study, further studies are needed with larger sample sizes. It is important to keep in mind that females generally have smaller brains than males (Ruigrok et al., 2014) and this can effect volume of specific brain regions (Pintzka et al., 2015). To control for this, TIV was entered as variable of no interest in all analyses. Furthermore, as the study is correlational in nature, any causal interpretations are unjustified.

## Conclusion

The present study documents opposing associations in males and females between temperament brain associations. The results reveal that sex-specific associations outweigh sex-general associations in the neurobiology of personality.

## Ethics Statement

This study was approved by the Ethical Committee of University Hospitals Leuven. All subjects gave written informed consent in accordance with the Declaration of Helsinki.

## Author Contributions

DS and JVdS contributed conception and design of the study and organized the database. DS performed the statistical analysis and wrote the first draft of the manuscript. Y-AH analyzed the data. All authors contributed to manuscript revision, read, and approved the submitted version.

## Conflict of Interest Statement

The authors declare that the research was conducted in the absence of any commercial or financial relationships that could be construed as a potential conflict of interest.

## Footnotes

1 All data is available through the corresponding author (jan.vandenstock@med.kuleuven.be).

2 http://www.neuro.uni-jena.de/cat/

3 http://www.fil.ion.ucl.ac.uk/spm/software/spm12/

4 http://www.alivelearn.net/xjview

5 http://www.mccauslandcenter.sc.edu/mricro/mricron
